# An Updated Study to Determine Association between Gadolinium-Based Contrast Agents and Nephrogenic Systemic Fibrosis

**DOI:** 10.1371/journal.pone.0129720

**Published:** 2015-06-15

**Authors:** Bin Zhang, Long Liang, Wenbo Chen, Changhong Liang, Shuixing Zhang

**Affiliations:** 1 Department of Radiology, Guangdong Academy of Medical Sciences/Guangdong General Hospital, Guangzhou, Guangdong Province, China; 2 Graduate College, Southern Medical University, Guangzhou, China; Hospital Universitario de La Princesa, SPAIN

## Abstract

**Background:**

Nephrogenic systemic fibrosis (NSF) is a rare but serious disorder disease affecting patients with advanced renal disease. Although multiple studies have indicated an association between gadolinium-based contrast agents (GBCAs) and NSF, some studies published after 2007 found no association. We therefore performed a meta-analysis to evaluate the association and analyze related (co)factors.

**Methods:**

Studies for analysis were identified by searching PubMed, Embase, and the Cochrane Central Register of Controlled Trials through December 2014. Pooled odds ratios (OR) with 95% confidence intervals (CI) were calculated using the fixed-effects model. Statistical heterogeneity was assessed by Q statistics and the I^2^ test. Publication bias was evaluated using Begg’s test, Egger’s test, funnel plot, and classic fail-safe N. Study quality was assessed using the Newcastle-Ottawa Scale. We also conducted sensitivity analyses, subgroup analyses and a cumulative meta-analysis. All statistical analyses were performed using Comprehensive Meta-Analysis 2.0.

**Results:**

A total of 14 studies (6,398 patients) met the inclusion criteria, but 3 were excluded since they reported no NSF events. Meta-analysis of controlled trials indicated a significant association between GBCAs and NSF development (OR = 16.504; 95% CI: 7.455–36.533; *P* < 0.001) and between gadodiamide and NSF (OR = 20.037; 95% CI: 3.725–107.784; *P* < 0.001). No statistical heterogeneity was observed across studies (*P* = 0.819, *I^2^* = 0%; *P* = 0.874, *I^2^* = 0%, respectively). Cumulative analysis demonstrated that the pooled ORs for association between GBCAs and NSF decreased post-2007 compared to pre-2007 (OR = 26.708; 95% CI: 10.273–69.436; *P*<0.001).

**Conclusions:**

Although this updated meta-analysis found a significant association between GBCAs and the incidence of NSF in patients with advanced renal disease, the association decreased after 2007. More studies, especially randomized controlled trials, are warranted to examine the potential association between GBCAs other than gadodiamide and NSF.

## Introduction

Nephrogenic systemic fibrosis (NSF), previously known as nephrogenic fibrosing dermopathy (NFD), is an idiopathic, progressive, systemic fibrosis disease that occurs in patients with renal diseases and can result in significant disability and even death [1,2]. Little is known about the etiology of NSF, and there is currently no consistently effective treatment [3]. Therefore, NSF prevention would be beneficial [4], ideally by confirming risk factors for the disease. Literature published prior to 2007 has not only suggested that free gadolinium, particularly gadodiamide, is a trigger of NSF [5], but has reported a strong causal relationship between gadolinium exposure and the development of NSF [6–18]; however, this association may be affected by other factors or cofactors, such as dosage or type of gadolinium, dialysis modality, renal disease severity, liver transplantation, chronic inflammation, or accelerated atherosclerosis [19–22].

In contrast, studies published after 2007 have reported few or even no NSF events in conjunction with gadolinium exposure and have therefore concluded a lack of association [23–28]. This change between studies conducted pre-2007 vs. post-2007 may be ascribed to a public health advisory issued by the United States (US) Food and Drug Administration (FDA) and guidelines released by the American College of Radiology (ACR) in 2007, as well as the class labeling of gadolinium requested by the European Medicines Agency (EMA) [29].

Here, we performed an updated meta-analysis to evaluate the association between gadolinium-based contrast agents (GBCAs) and NSF pre- and post- 2007 and attempted to analyze relevant factors or cofactors that may have influenced this association.

## Materials and Methods

This study was reported in accordance with the Preferred Reporting Items for Systematic Reviews and Meta-Analyses (PRISMA) statement (S1 File) [30].

Two authors (B.Z. and L.L.) independently extracted data. Disagreements were solved by negotiation, consensus, and decision by a third author (WB.C). As this was a meta-analysis that did not involve identifiable patient data, no particular ethical considerations were required.

### Literature search and study selection

First, we identified relevant studies via electronic searches of PubMed, Embase, and the Cochrane Central Register of Controlled Trials (CENTRAL) from inception to December 2014 using the following key words and/or medical subject headings (MeSH) singly and in combination: GBCA, gadolinium, contrast media, contrast agent, magnetic resonance imaging, MRI, nephrogenic systemic fibrosis, nephrogenic fibrosing dermopathy, NSF, and NFD. The detailed search strategy can be found in the supporting information (S2 File). A search of all related studies’ references was also performed. Included studies were required to be human studies, and no language limitations were imposed. Duplicate publications were removed.

In our second step, we screened the titles and abstracts of papers identified in the first step. Those that met our inclusion criteria were deemed eligible for full-text review. The main inclusion criterion was that the study examined association between GBCAs and NSF; comments, letters, review publications, and irrelevant studies were excluded.

Third, we reviewed the literature screened by the second step. Studies were further excluded for the following reasons: no control group, unable to extract data, and nonoriginal research.

### Data extraction and quality assessment

The outcome of interest was the incidence of NSF/NFD in association with gadolinium exposure. Baseline characteristics extracted from all studies included country, study design, study time span, population history, GBCA type used, outcome measure, and study results. We also determined whether a dose-response relationship between GBCAs and NSF was reported. For quality assessment of included studies, the Newcastle-Ottawa Scale for cohort studies and case-control studies was used [31]. The scale for cohort studies encompassed 3 main items: (1) Selection, (2) Comparability, and (3) Outcome. The scale for case-control studies also consisted of 3 items: (1) Selection, (2) Comparability, and (3) Exposure. After these 3 main items, the scales included 8 subitems. All scores are represented out of 9 possible stars (Table 1). A higher score indicates that the individual study was of higher quality. Discrepancies in the score were resolved through discussion between the authors.

**Table 1:** Checklist for quality assessment of cohort and case-control study.

*Cohort study*
**Selection**
1. How representative was the GBCAs group compared with the general population exposed to
GBCAs? (if yes, one star, no star if the patients were selected)
2. Was the source of non-exposed cohort same as exposed cohort? (if yes, one star, no star if
drawn from a different source or the source was not described)
3. Ascertainment of exposure: any reliable document or criteria reported (if yes, one star)
4. Exclusion of outcome of interest at start of study (if yes, one star)
**Comparability**
5. Adjustment for confounding factors? (if age- and sex-matched, one star, other important factors
controlled, one star)
**Outcome**
6. Was outcome obtained via rigorous assessment? (if yes, one star)
7. Was follow up long enough for outcome to occur? (if yes, one star)
8. Low loss to follow up of cohorts (if yes, one star)
***Case-control study***
**Selection**
1. Was the Case Definition Adequate? (e.g. hospital records) (if yes, one star, no star if definition
was inadequate or definition was not described)
2. Was the case collected consecutively and representative? (if yes, one star)
3. Was the source of control group same as case group? (if yes, one star, no star if drawn from a
different source or the source was not described)
4. Were controls had no history of this outcome(endpoint)and definited explicitly? (if yes, one
star)
**Comparability**
5. Adjustment for confounding factors? (if age- and sex-matched, one star, other important factors
controlled, one star)
**Exposure**
6. Ascertainment of Exposure: any reliable document or others? (if yes, one star)
7. Same method of ascertainment for cases and controls? (if yes, one star)
8. Was non-response rate same in both groups? (if yes, one star)

### Data synthesis and analysis

Data synthesis and analysis were performed using the fixed-effects model (Mantel- Haenszel method) with Comprehensive Meta-Analysis V2 (Biostat, Englewood, NJ, United States). Studies that did not report any NSF cases were excluded from the final meta-analysis [32]. For each study, we calculated the odds ratio (OR) with 95% confidence interval (CI). A 2-tailed P-value <0.05 was considered significant. We assessed statistical heterogeneity between 2 studies using Cochrane’s Q test (*P* < 0.10 considered significant) and I^2^ statistics (25%, 50%, and 75% represented low, moderate, and high heterogeneity, respectively). Publication bias was evaluated using Begg’s test (rank regression), Egger’s test (linear regression), and funnel plot, except in cases where included studies were <10 [33]. Rosenthal’s fail-safe N test was used to compute the number of missing studies (with a mean effect of zero) that would need to be added to the analysis to yield an overall nonsignificant effect (*P* > 0.05). A higher N meant more robust results. In addition, sensitivity analysis was conducted by omitting 1 study at a time to determine if any single study excessively influenced the summary estimates. Cumulative analysis was applied to compare pooled ORs of all studies published chronologically to a specific point in time. Subgroup analyses were used to determine some possible impacts on the association between GBCAs and NSF, such as age or gender, country studied, sample size, medium contrast dose, and type of study.

## Results

### Study flow diagram and baseline characteristics

**Fig 1:**
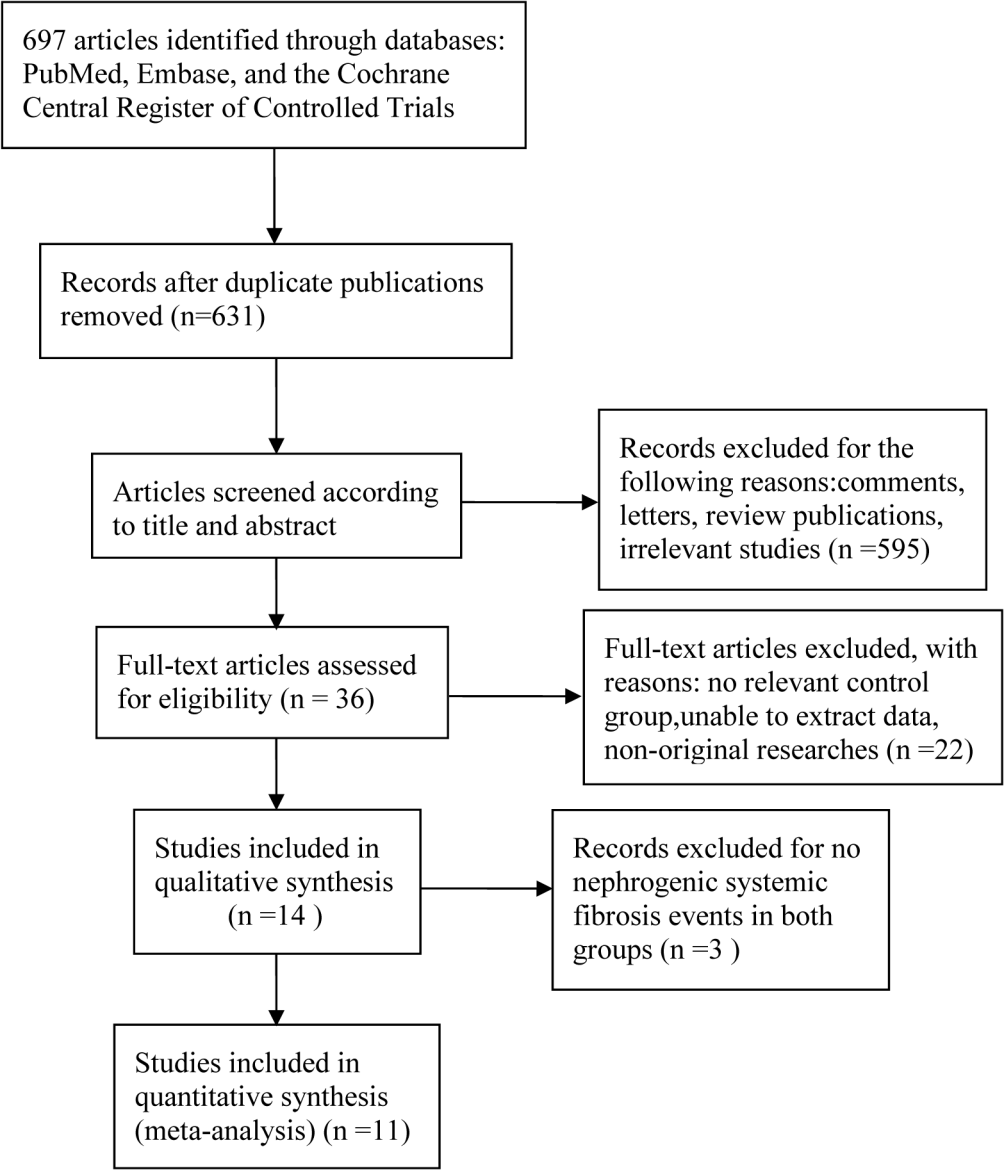
Flow diagram of included studies.

Our literature search obtained 697 publications. Of these, 683 were excluded due to result duplicity, irrelevant to the current analysis, nonoriginal research, or insufficient data for analysis; therefore, 14 studies met the initial inclusion criteria to be enrolled into this study. A total of 3 studies (published in 2010, 2013, and 2014, respectively) were excluded from the final meta-analysis due to no reports of NSF events. The remaining 11 studies were analyzed (Fig 1).

The baseline characteristics of included studies are shown in Table 2. We identified 12 cohort studies and 1 case-control study, but no randomized controlled trials. The initial 14 studies included a total of 6,398 patients with advanced renal disease that required dialysis. The 11 studies analyzed in the final meta-analysis included a total of 5,405 patients. All studies were performed in the US or in Europe. The time spans of the studies ranged from 3 months to 12 years. Gadodiamide was the sole contrast agent used in 3 studies [9,12,13]. Two studies injected meglumine gadoterate (Gd-DOTA) and gadopentetate, respectively [8,25]. A diagnosis of NSF depended on clinical examinations and skin biopsies except for 1 study which only used clinical examinations [8].The results from 9 of these studies showed an association between gadolinium exposure and NSF, while 4 did not [23–26].

**Table 2:** The baseline characteristics of included studies.

Study	Country	Design	Time span	History ofpopulation	GBCAs	Diagnosis of NSF	Results
Marckmann	Denmark	①	10mo	ESRD	Gadodiamide (Omniscan)	clinical examinations, skin biopsy	+$
Broome	US	①	>6yr	renal insufficiency	Gadodiamide (Omniscan)	clinical examinations, laboratory findings, skin biopsy	+$
Deo	US	①	18mo	ESRD	NA	clinical examinations, skin biopsy	+$
Othersen	US	①	6yr	CKD	Gadodiamide (Omniscan)	clinical examinations, skin biopsy	+$
Todd	US	②	2yr	renal disease	Gadopentetate (Magnevist)	clinical examinations	+$
Collidge	Scotland	①	6.5yr	renal disease	Multiple‡	clinical examinations, laboratory findings, skin biopsy	+$
Wiginton	US	①	10yr	renal failure	Gadodiamide/ Gadopentetate	clinical examinations, laboratory findings, skin biopsy	+$
FINEST	France	①	1yr	renal impairment	Multiple‡‡	clinical examinations, skin biopsy	_♯
Heinz-peer	Austria	①	10yr	ESRD	Multiple†	clinical examinations, skin biopsy	+$
CDC	US	③	>6yr	renal disease	NA	clinical examinations, skin biopsy	+$
RESCUE	Belgium/France/Italy/Spain	②	>3mo	CKD	Gd-DOTA	clinical examinations, laboratory findings, skin biopsy	_♯
Becker	German	①	4yr	ESRD	Multiple*	clinical examinations, skin biopsy	_♯
Elmholdt	Denmark	③	12yr	renal insufficiency	Multiple¶	clinical examinations, skin biopsy	+$
Amet	France	②	>2yr	renal insufficiency	Multiple**	clinical examinations, skin biopsy	_♯

Note: ①/②/③represented retrospective cohort study, prospective cohort study, case-control study, respectively.

mo = months, yr = years.ESRD = end stage renal disease,CKD = chronic kidney disease,GBCAs = gadolinium-based

contrast agents, NSF = nephrogenic systemic fibrosis, NA = not available, Gd-DOTA = meglumine gadoterate

‡88.6% MR examinations were performed with gadodiamide (Ominscan), 11.4% were performed with Multihance, Magnevist,etc.

‡‡76% received gadoterate

†Seven types of GBCAs were used in MR examinations

* Five types of GBCAs

¶ Gadodiamide, gadopentetate dimeglumine, gadobutrol, etc.

**Seven types of GBCAs

$ Positive association was found between GBCAs and NSF

♯ Insufficiency evidence showed GBCAs was associated with the development of NSF.

### Decreased association between all GBCAs and NSF after 2007

**Fig 2:**
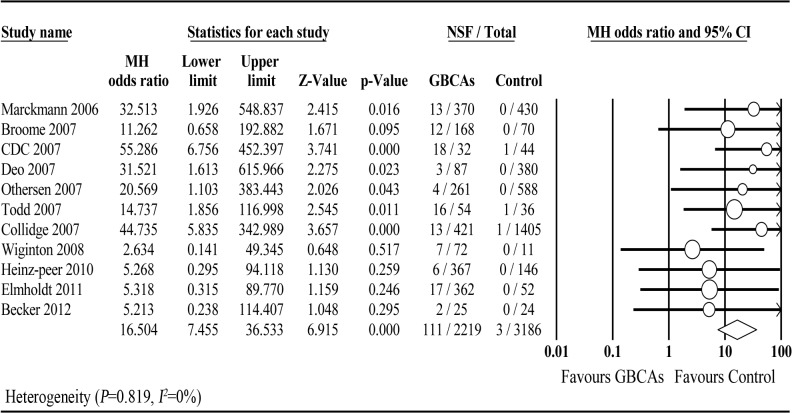
Forest plot of association between GBCAs and the development of NSF.

**Fig 3:**
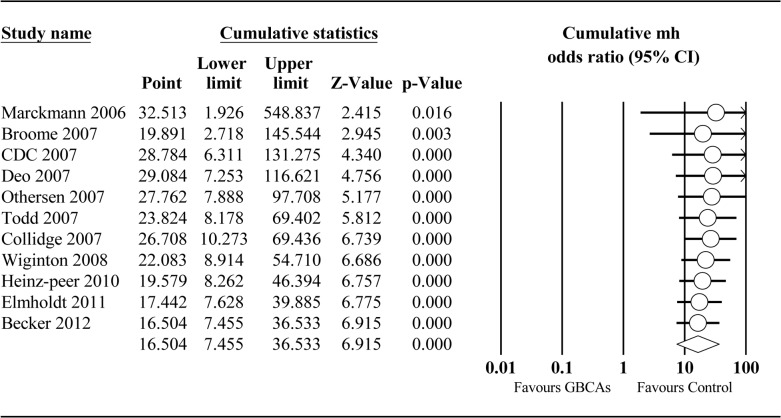
Cumulative meta-analysis plot of GBCAs group vs Control group. We added one study at a time chronologically until all studies were included.

Our meta-analysis demonstrated an obvious association between GBCAs and NSF (OR = 16.504; 95% CI: 7.455–36.533; *P* < 0.001) (Fig 2). No significant heterogeneity was observed across studies (*P* = 0.819, *I* ^*2*^ = 0%). Evidence from the cumulative analysis indicated a decrease in the risk of NSF linked to GBCA exposure post-2007 compared with pre-2007 (OR = 26.708; 95% CI: 10.273–69.436; *P* < 0.001) (Fig 3).

**Fig 4:**
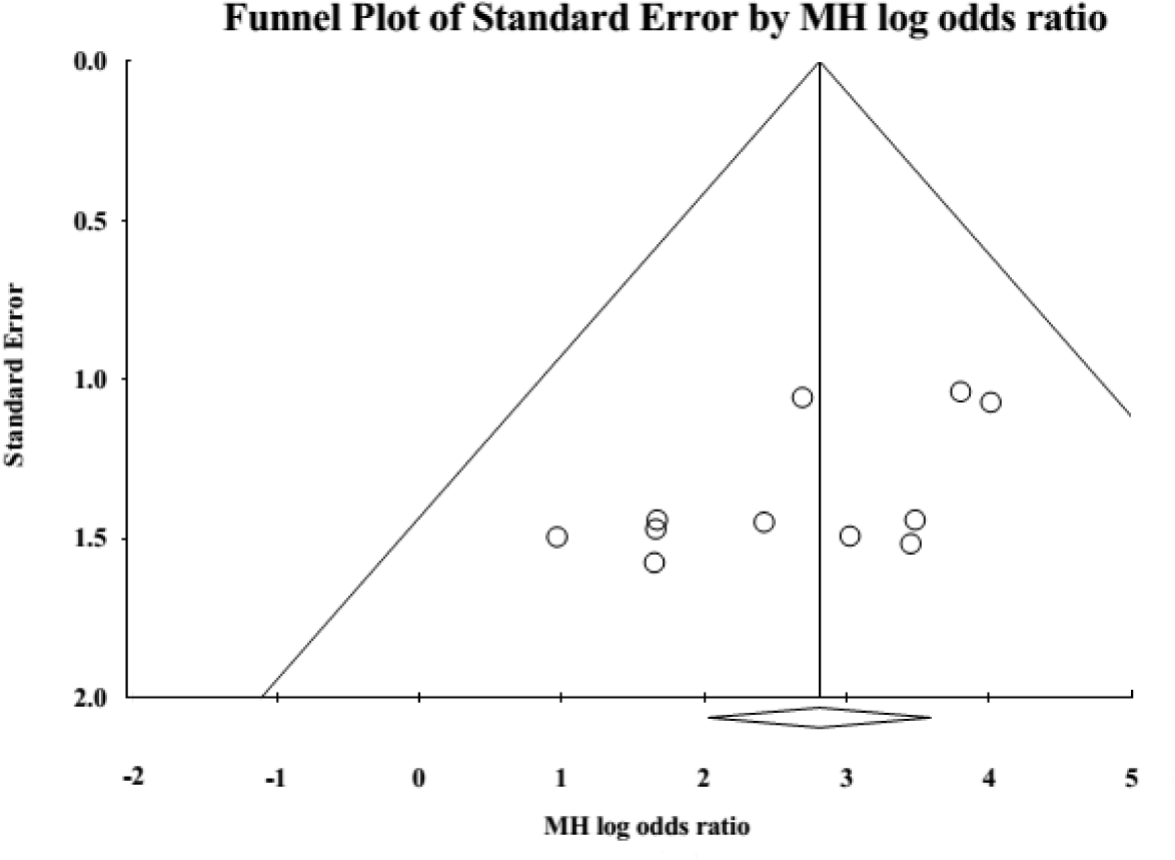
Funnel plot for publication bias.

**Fig 5:**
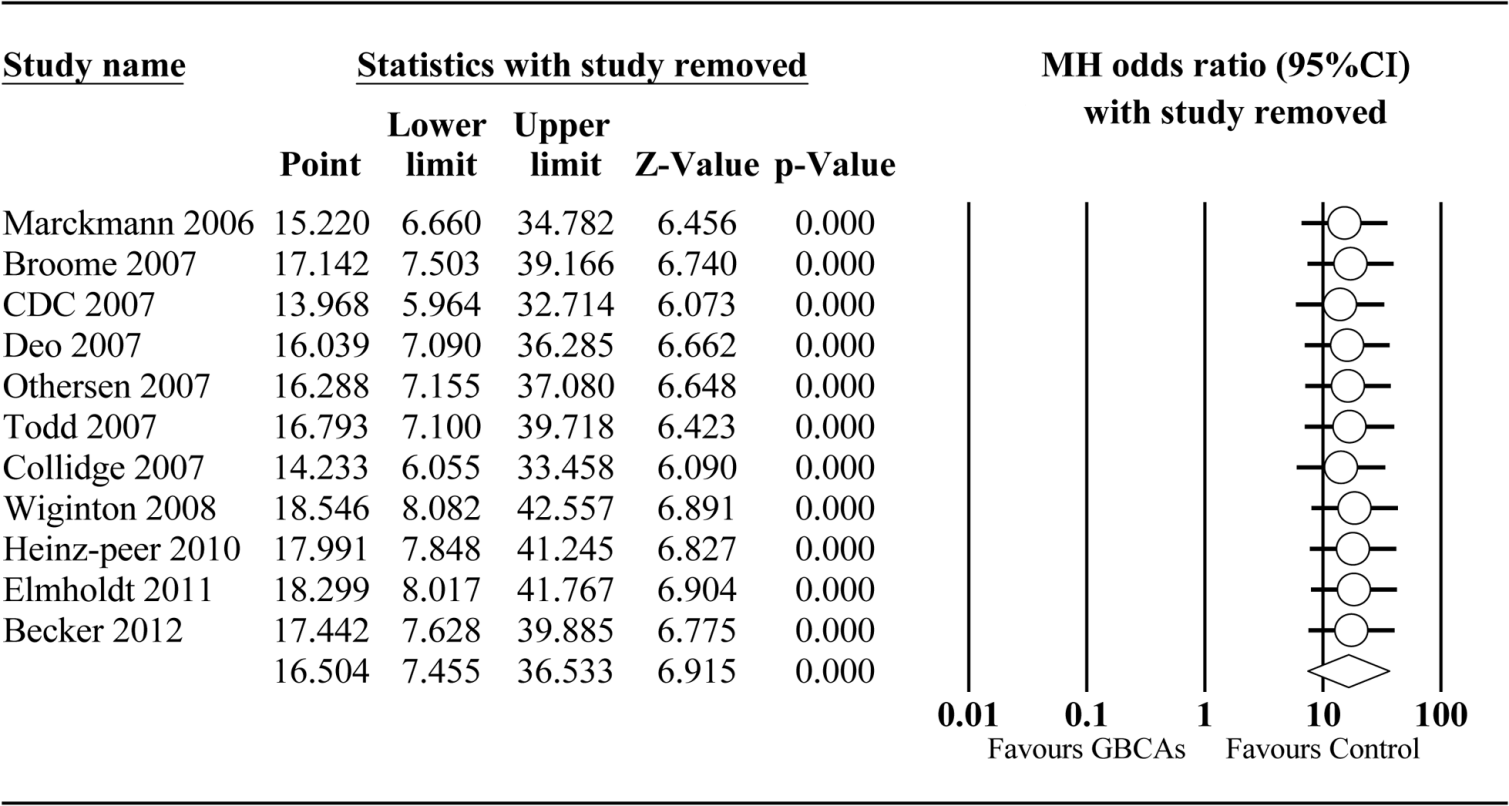
Sensitivity analysis plot of GBCAs group vs Control group. To assess the influence of single study on pooled estimate, we omitted one study at a time.

1. **Publication bias.** Asymmetry was observed upon visual inspection of funnel plots (Fig 4); however, quantitative assessment by Begg’s test (*P* = 0.186; continuity correction *P* = 0.213) and Egger’s test (*P* = 0.042) suggested that there was little publication bias. Rosenthal’s fail-safe N was 119, meaning that 119 ‘null’ studies would need to be located and included in order for the combined 2-tailed *P*-value to exceed 0.05. Therefore, the result was relatively robust.
2. **Quality assessment.** The median Newcastle-Ottawa quality score for the included studies was 6 stars (range, 5–8 stars). Only 2 studies controlled for confounding factors [14,34] (Table 3).
3. **Sensitivity analysis.** The forest plot of sensitivity analysis indicated that no single study had an excessive influence on the pooled analysis (Fig 5).
4. **Subgroup analysis.** We couldn’t perform subgroup analyses according to age or gender, medium contrast dose, and type of study due to unknown or insufficient data. Subgroup analysis by country showed no significant difference between the US and Europe countries (OR = 17.35; 95% CI: 5.98–50.33; *P* < 0.001 vs OR = 15.59; 95%CI: 4.75–51.18; *P* < 0.001) (Table 4). However, a marked difference was observed by sample size (OR = 13.32; 95% CI: 4.93–35.99; *P* < 0.001 vs OR = 23.41; 95%CI: 6.24–87.88; *P* < 0.001) (Table 4).

**Table 3:** Quality assessment of included studies.

Author (year)	Selection				Comparability	Outcome† or Exposure‡			Total score
	1	2	3	4	5	6	7	8	
Marckmann 2006	-	☆	☆	☆	—	☆	☆	☆	☆☆☆☆☆☆
Broome 2007	-	☆	☆	☆	—	☆	☆	☆	☆☆☆☆☆☆
Deo 2007	-	☆	☆	☆	—	☆	☆	☆	☆☆☆☆☆☆
Othersen 2007	-	☆	☆	☆	—	☆	☆	☆	☆☆☆☆☆☆
Todd 2007	-	☆	☆	☆	—	-	☆	☆	☆☆☆☆☆
Collidge 2007	-	☆	☆	☆	—	☆	☆	☆	☆☆☆☆☆☆
Wiginton 2008	-	☆	☆	☆	—	☆	☆	☆	☆☆☆☆☆☆
CDC 2007‡	-	☆	☆	☆	☆	☆	☆	☆	☆☆☆☆☆☆☆☆
Heinz-peer 2010	-	☆	☆	☆	—	☆	☆	☆	☆☆☆☆☆☆
FINEST 2010	-	☆	☆	☆	—	☆	☆	☆	☆☆☆☆☆☆
Elmholdt 2011‡	-	☆	☆	☆	☆	☆	☆	-	☆☆☆☆☆☆
RESCUE 2013	-	☆	☆	☆	—	☆	-	☆	☆☆☆☆☆
Becker 2012	-	☆	☆	☆	—	☆	☆	☆	☆☆☆☆☆☆
Amet 2014	-	☆	☆	☆	—	☆	☆	☆	☆☆☆☆☆☆

Note:—no star † cohort study ‡ case-control study

**Table 4:** Subgroup analyses on the incidence of NSF in various conditions.

Conditions	Included study	OR with 95%CI	Heterogeneity analysis
Country			
Europe	5	15.59 (4.75–51.18; *P*<0.001)	*I* ^*2*^ = 0%; *P* = 0.579
US	6	17.35 (5.98–50.33; *p*<0.001)	*I* ^*2*^ = 0%; *P* = 0.694
Patients			
< 500	7	13.32 (4.93–35.99; *p*<0.001)	*I* ^*2*^ = 0%; *P* = 0.671
> 500	4	23.41 (6.24–87.88; *p*<0.001)	*I* ^*2*^ = 0%; *P* = 0.688

### Association between gadodiamide and NSF

**Fig 6:**
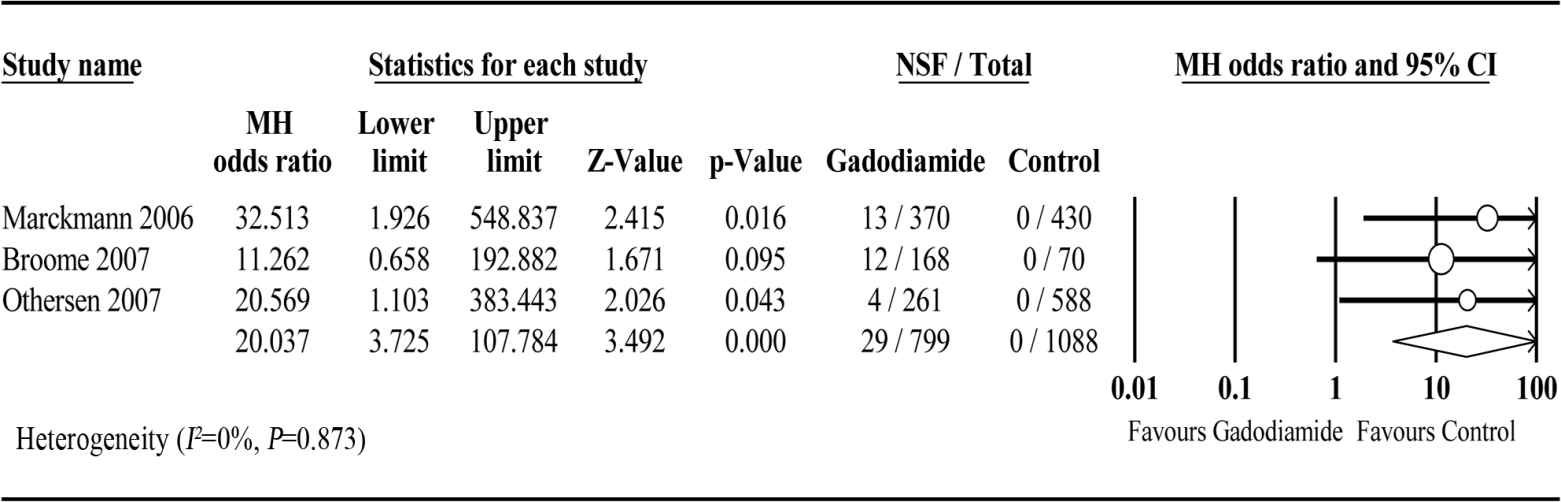
Forest plot of association between Gadodiamide exposure and the development of NSF.

**Fig 7:**
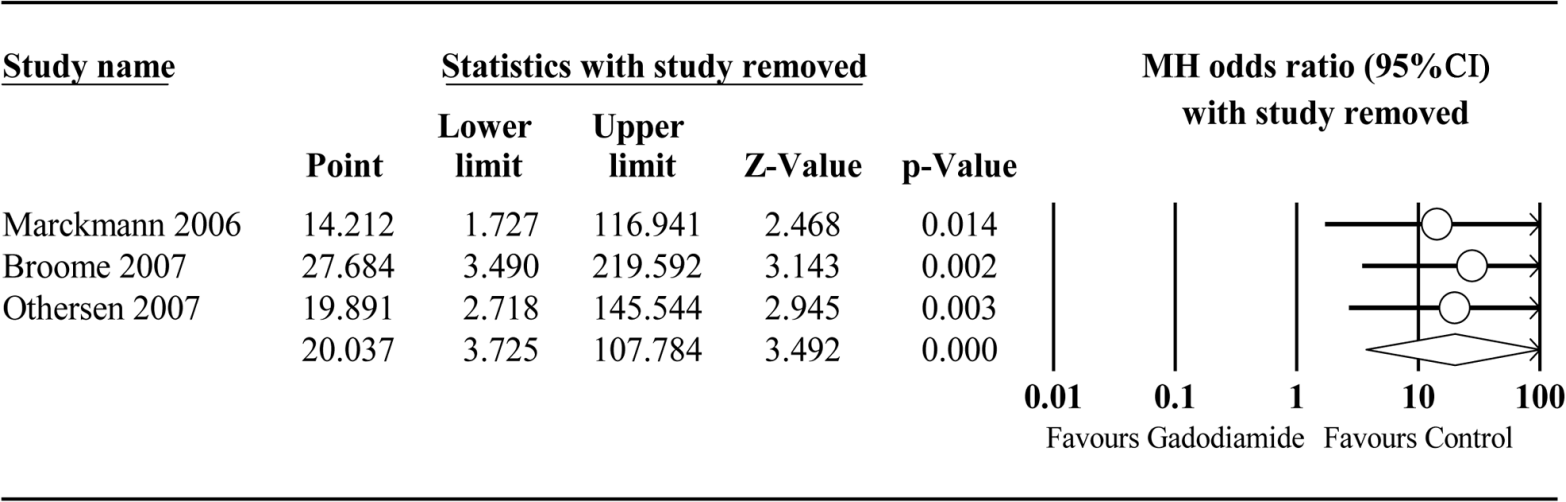
Sensitivity analysis plot of gadodiamide group vs. control group. To assess the influence of single study on pooled estimate, we omitted one study at a time.

To date, only 3 studies exclusively injected gadodiamide in patients with renal disease. A meta-analysis of these 3 studies revealed a significant link between gadodiamide and NSF (OR = 20.037; 95% CI: 3.725–107.784; *P* < 0.001). No significant heterogeneity was observed across studies (*P* = 0.873, *I* ^*2*^ = 0%) (Fig 6). Since the number of included studies was less than 10, the power of publication bias evaluation was very low and therefore was not assessed. The Newcastle-Ottawa quality scores of all studies were 6 stars (Table 3). No single study excessively affected the pooled estimates (Fig 7).

### Association between other GBCAs besides gadodiamide and NSF

Within the eligible studies, 2 studies reported an injection of Gd-DOTA and gadopentetate, respectively [8,25]. The OR for NSF in the study that administered gadopentetate was 14.737 (95% CI = 1.856–116.998; *P* = 0.011). No NSF events were reported in the study that used Gd-DOTA, making determination of its association with NSF impossible. There were no other controlled or uncontrolled studies determining the association between other GBCAs and NSF.

### Dose-response relationship between GBCAs and NSF

In total, 4 studies described a dose-response relationship between gadodiamide exposure and the risk of NSF [7,10,12,34] (Table 5). Collidge et al reported that patients with NSF received a higher median cumulative dose of gadodiamide than their unaffected gadolinium-exposed counterparts (0.39 vs. 0.23 mmol/kg of body weight, respectively, *P* = 0.008) [10]. The other 3 studies also showed gadodiamide dosage differences between patients with and without NSF.

**Table 5:** Dose-response relationship between gadolinium and NSF.

Study	Year	Gadolinium dose		OR(95%CI)	*P* value
		NSF group	Non-NSF group		
Collidge	2007	0.39*	0.23*	NA	.008
Broome	2007	0.20†	0.10†	12.1(0.7–206.2)	NA
Heine-peer	2010	59.5‡	25.0‡	NA	.028
Elmholdt	2011	57±36※	25±13※	NA	.012

Note: NA = not applicable

* cumulative dose, median (mmol/kg)

†mean (mmol/kg)

‡median (ml)

※ mean±sd(ml)

## Discussion

### Main findings and analysis

Our updated meta-analysis suggested a significant association between GBCA exposure and the development of NSF, consistent with another systematic review and meta-analysis conducted pre-2007 [35]. Nevertheless, our cumulative meta-analysis indicated an obvious decrease in the association between GBCA exposure and NSF post-2007. If the “zero case effect” of 3 of the included studies is considered, the decreased association between GBCAs and NSF post-2007 may be overestimated.

From 1997 to 2007, more than 500 NSF cases were reported in patients with severe renal insufficiency (glomerular filtration rate <30 ml/min/1.73 m^2^) [36]. The FDA and ACR both issued warnings against the use of gadolinium in this renal function-compromised population due to the suspected link. Our findings supported these warnings. All NSF cases in our study occurred in patients with severe renal disease. The FDA and EMA also requested that manufacturers of all approved GBCAs, drugs widely used for magnetic resonance imaging, use nearly identical text in their product labeling (revised in 2010) to describe the risk of NSF [29]. Evaluation of renal function (e.g. serum creatinine level) and history was also recommended before use of GBCAs. As a result, the prevalence of NSF has decreased dramatically [37–39]. Our cumulative meta-analysis also found a decreased effect and may reflect these changes in policy.

Other factors or cofactors may affect the association between GBCAs and NSF. In our meta-analysis, 4 studies described a dose-response relationship between gadodiamide and NSF. Broome et al. showed that the risk of NSF was 12.1 times higher in a double dose group compared with a single dose group [12]. Other studies reported similar findings [40,41]. Use of higher gadolinium doses than were recommended by regulatory agencies contributed to NSF [42]. Additionally, certain chelates were shown to be risk factors for NSF development after GBCA exposure [43]. GBCAs are classified according to their chemical structure as macrocyclic vs. linear, or ionic vs. nonionic. In contrast to ionic, macrocyclic chelates, nonionic, linear chelates bind gadolinium less tightly, resulting in a less stable gadolinium-ligand complex with higher free gadolinium dissociation rates [43,44]. For this reason, researchers hypothesized that nonionic, linear chelates (e.g. gadodiamide, gadoversetamide) are more likely to cause NSF than ionic macrocyclic chelates (e.g. dotarem) [20,45]. In our study, we did not find published reports examining the link between GBCAs other than gadodiamide/gadopentatate and NSF.

Other risk factors or cofactors may play a concomitant role in NSF development. A case-control study from Denmark showed that of 17 NSF cases, 41% received hemodialysis, 24% peritoneal dialysis, and 35% no dialysis [34]. The authors concluded that the risk of NSF may be higher in patients who underwent hemodialysis than in patients who underwent peritoneal dialysis. The NSF risk was also strongly correlated with the severity of renal disease, measured by renal excretion, which determines the duration of GBCA exposure and therefore the extent of gadolinium ion release. Almost all NSF cases develop in dialysis patients with severe to end-stage (stage 4–5) chronic kidney disease (CKD). Very few cases of NSF are seen in patients with a glomerular filtration rate >30 ml/min [46]. Cofactors such as chronic inflammation and accelerated atherosclerosis seem to be involved in the development of NSF, but their role is still unclear [21].

To reduce the risk of NSF, gadolinium exposure and other risk factors and cofactors should be controlled by physicians. Researchers have conducted trials that limit GBCA to the recommended dose or even to a quarter-dose (0.025 mmol/kg), that dialyze patients quickly following GBCA administration, and that avoid using nonionic, linear GBCA in patients with renal failure especially when there are proinflammatory and/or atherosclerotic conditions may significantly reduce the risk of NSF[47,48].

### Limitations

There were some limitations of this meta-analysis. First, no randomized controlled trials were available for inclusion, and most of the included studies did not adjust for confounding factors. Second, the authors were unable to agree upon a method to calculate the effect of the 3 studies that reported “zero NSF cases”. Third, since all included studies were performed in either the US or Europe, the summary estimates may not be applicable to other countries, though the result of subgroup analysis by country showed no significant difference between the US and Europe countries. Fourth, since almost all patients in our study had stages 3–5 of CKD, we are unable to comment on the risk of NSF in patients with acute renal failure or other stages of CKD. Lastly, we could not find any controlled or uncontrolled published studies examining the association between NSF and GBCAs in forms other than gadodiamide.

## Conclusions

This study demonstrates a significant association between GBCAs and NSF; however, the association decreased in studies reported after 2007, likely a result of policies issued by the FDA and ACR. The association can be affected by the GBCA dose, GBCA chemical structure, dialysis modality, renal disease severity, proinflammatory and/or atherosclerotic conditions, and other factors. Thus, clinicians should minimize gadolinium exposure and carefully assess risks and benefits, especially for patients with severe to end-stage CKD. These recommendations are in line with the FDA and ACR guidelines.

## Supporting Information

S1 File PRISMA Checklist (DOC). (DOC)

S2 File Search strategy S2 (DOC). (DOCX)
