# Supplementary material for: An Updated Study to Determine Association between Gadolinium-Based Contrast Agents and Nephrogenic Systemic Fibrosis
Source: PLoS One. 2015 Jun 15;10(6):e0129720. doi: 10.1371/journal.pone.0129720 (PMC4468111; doi:10.1371/journal.pone.0129720)
Supplement: S2 File — (DOCX) [file pone.0129720.s002.docx]

*Search strategy*

*PubMed*

# 1 “nephrogenic fibrosing dermopathy” [MeSH Terms]

# 2 “nephrogenic systemic fibrosis”

# 3 “NSF”

# 4 “NFD”

# 5 # 1 OR # 2 OR # 3 OR # 4

# 6 “gadolinium” [MeSH Terms]

# 7 “gbca” [MeSH Terms]

# 8 “contrast media”[MeSH Terms]

# 9 “contrast agent”[MeSH Terms]

# 10 # 6 OR # 7 OR # 8 OR # 9

# 11 “magnetic resonance imaging”[MeSH Terms]

# 12 “mri”[MeSH Terms]

# 13 #11 OR #12

#14 #5 AND #10 AND #13

# 15 #14 AND humans [MeSH Terms]

*Cochrane Central Register of Controlled Trials (CENTRAL)in The Cochrane Library*

# 1 MeSH descriptor: [nephrogenic fibrosing dermopathy] explode all trees

# 2 (nephrogenic systemic fibros* OR nephrogenic fibrosing dermopath* OR "NSF" OR "NFD")

# 3 (#1 OR #2)

# 4 MeSH descriptor: [gadolinium] explode all trees

# 5 (GBCA* OR "contrast media" OR "contrast agent" OR Gd*)

# 6 (#4 OR #5)

# 7 MeSH descriptor: [magnetic resonence imaging] explode all trees

# 8 MeSH descriptor: [mri] explode all trees

# 9 (#7 OR #8)

# 10 (# 3 AND #6 AND #9)

Note: the search strategy of Embase was similar to that of PubMed

窗体顶端

窗体底端
